# Shift work and its association with metabolic disorders

**DOI:** 10.1186/s13098-015-0041-4

**Published:** 2015-05-17

**Authors:** Maria Carlota Borba Brum, Fábio Fernandes Dantas Filho, Claudia Carolina Schnorr, Gustavo Borchardt Bottega, Ticiana C. Rodrigues

**Affiliations:** Division of Occupational Medicine, Hospital de Clínicas de Porto, Alegre, Rio Grande do Sul Brazil; Post-graduate program in Medical Sciences, Endocrinology, Universidade Federal do, Porto Alegre, Rio Grande do Sul Brazil; Department of Internal Medicine, Medical School, Universidade Federal do, Porto Alegre, Rio Grande do Sul Brazil; Division of Endocrinology, Hospital de Clínicas de Porto Alegre, Rua Ramiro, Barcelos 2350, Prédio 12, 4° andar, Porto Alegre, Rio Grande do Sul 90035-003 Brazil

**Keywords:** Shift work, Obesity, Metabolic syndrome, Diabetes, Hypertension, Sleep restriction, Insulin resistance

## Abstract

Although the health burden of shift work has not been extensively studied, evidence suggests that it may affect the metabolic balance and cause obesity and other metabolic disorders. Sleep deprivation, circadian desynchronization and behavioral changes in diet and physical activity are among the most commonly mentioned factors in studies of the association between night work and metabolic disorders. Individual adaptation to night work depends greatly on personal factors such as family and social life, but occupational interventions may also make a positive contribution to the transition to shift work, such as exposure to bright lights during the night shift, melatonin use, shift regularity and clockwise rotation, and dietary adaptations for the metabolic needs of night workers. The evaluation of the impact of night work on health and of the mechanisms underlying this relationship can serve as a basis for intervention strategies to minimize the health burden of shift work. This review aimed to identify highlights regarding therapeutic implications following the association between night and shift work and metabolic disorders, as well as the mechanisms and pathways responsible for these relationships.

## Introduction

Twenty-four hour services are a growing part of modern society. Essential services are provided without interruption, and several industries and business establishments operate on a 24 h basis so as to meet the constantly changing demands of the modern world [1]. As a result, companies require employees to work continuously, creating a need for shift- and night-work schedules.

Shift schedules allow companies to operate on a continuous basis by ensuring that positions are always filled by rotating employees. Night work is defined as a work shift lasting at least seven consecutive hours, which comprehends the interval between midnight and five o'clock in the morning [2].

Shift frequency and duration may vary between companies, and differences may also be observed in the number of consecutive work days and the direction of rotation [3]. Although no studies on the topic have been published in Brazil, European research has reported that 20 % of workers in the continent perform shift or night work [4].

The growing importance of shift and night work in meeting the demands of modern society creates an urgent need for research into the effects of such schedules on worker health.

The effects of shift work on health have only been sparsely studied, but recent findings suggest that such schedules may affect glucose tolerance and induce obesity and systemic arterial hypertension (SAH) [5–8].

This review aimed to identify highlights regarding some therapeutic implications following the association between night and shift work and metabolic disorders, as well as the mechanisms and pathways responsible for these relationships. Metabolic syndrome, diabetes, obesity and cardiovascular disease are conditions with recent association with night work.

### Shift work and its effects on health

#### Cardiovascular

According to a recent meta-analysis, shift work is associated with an increased risk of coronary disease, acute myocardial infarctions and cerebrovascular accidents, even after adjusting for possible confounds. However, shift work was not associated with overall mortality, or cardiac or cerebrovascular death [9].

A study performed in a public university in Brazil evaluated cardiovascular risk in 211 workers of both genders aged between 30 and 64 years using Framingham scores, and found that both cardiovascular risk (28 %) and the prevalence of SAH (33.4 %) were higher in night workers than in day workers [10].

A study of worker health performed in seven French hospitals evaluated participants on two occasions and found that the systolic arterial pressure of night workers was 2.5 mmHg (*p* < 0.001) higher than that of day workers [11].

A British study which followed a cohort of 7839 participants from birth until age 45 found that cardiovascular risk, body mass index (BMI), abdominal circumference, total cholesterol, triglyceride levels, glycated hemoglobin and C-reactive protein levels were higher in men who performed shift work on a regular basis than in those who did not usually work in shifts. In women, shift work was positively associated with triglyceride levels, but negatively associated with diastolic arterial pressure. Adjusting for confounders (alcohol consumption, exercise, tobbaco and consumption fruits, vegetables and fried foods) had little impact on results regarding adiposity measures, but attenuated the association between shift work and the levels of cholesterol, triglyceride, glycated hemoglobin and inflammatory markers. After this adjustment, only BMI, abdominal circumference and C-reactive protein levels were still found to be higher in night workers [12]. Therefore, this large cohort shows that health habits can reduce the negative effect of shift work in cardiovascular risk.

A Japanese study compared the annual checkups of day workers with those of adults with alternating day/night shifts between 1991 to 2005. Alternating shift workers were found to have increased arterial pressure, even after adjusting for confounding factors. These findings suggested that alternating shift work may constitute an independent risk factor for increased blood pressure levels regardless of the known risk factors such as age and BMI [13].

Given the decrease in sleep duration observed over the past century, there is also a need for research into the association between sleep duration and occurrence of SAH and obesity. Currently, over 30 % of adults in the United States aged between 30 and 64 years sleep less than 6 h per night. Decreased sleep duration is often accompanied by a significant increase in the prevalence of obesity and SAH [14].

The First National Health and Nutrition Examination Survey (NHANES I) evaluated the relationship between sleep and the incidence of SAH in American adults. The incidence of SAH was associated with a sleep duration of 5 h or less (OR: 2.10; 95 % CI 1.58 - 2.79) in subjects aged 32 to 59 years. The control of confounding factors only attenuated this relationship. The risk of SAH in subjects with reduced sleep duration remained significantly elevated after adjusting for the presence of obesity and diabetes. This finding was consistent with the hypothesis that these conditions may act as partial mediators in the relationship between hypertension and sleep duration. The study concluded that sleep deprivation in healthy subjects can increase blood pressure levels and sympathetic nervous system activity [15]. Chronic short sleep duration may also contribute to the development and maintenance of SAH by inducing a prolonged increase in blood pressure levels and heart rate, increasing sympathetic activity, imposing physical and psychosocial stressors and increasing salt retention. Prolonged exposure to these factors may entrain the cardiovascular system to operate at an elevated pressure equilibrium through structural adaptations such as left ventricular hypertrophy [15].

### Obesity/overweight

The association between weight-gain and night or shift work has also been investigated. A study performed in southern Brazil reported especially high rates of obesity in female night workers aged 40 years or older with low education levels and a family history of overweight. In this study, daily sleep duration was also divided into the three following categories: >5 h of continuous sleep, ≤5 h of continuous sleep with some additional rest, or ≤5 h of continuous sleep with no additional rest. After adjusting for confounding factors, obesity rates were found to be significantly higher in the latter two groups than in the first (composed largely of day workers), supporting the association between sleep deprivation and obesity [16].

A Japanese study which followed 21,693 men and 2109 women between 1999 and 2006 found that the relative risk of obesity was higher in men who slept for less than 5 h/24 h than in those who had at least 5 to 7 h of sleep per day. In women, these variables were not significantly associated. The study concluded that short sleep durations (<5 h) accelerated the onset of obesity in shift workers [17].

A systematic review performed by Van Drongelen [18] provided strong evidence of the association between shift work and increased body weight. Furthermore, behavioral changes potentially associated with shift work, such as reduced physical activity, may independently contribute to weight gain and the development of associated conditions such as metabolic syndrome and type 2 diabetes. However, the generalization of these findings is limited by the heterogeneity of the studies included in the meta-analysis, which varied widely in follow-up methods and periods, in the control of confounding factors, and in their definitions of shift work.

In a study performed by Di Lorenzo et al. [19], obesity was more prevalent in shift workers (20.0 %) than in day workers (9.7 %). Shift work was found to be associated with BMI regardless of age or duration of shift work exposure.

Despite all the evidence exposed above show great strength in the association between shift work and obesity, the mechanisms responsible for the association between these factors cannot yet be fully explained [20, 21] and more studies are needed to understand the pathogenesis of this association.

### Metabolic syndrome

A 5 year follow-up study of 387 female employees in Taiwan performed by Cheng Lin et al. [22] found that rotating shift work had significant deleterious effects on health, and resulted in an increased risk of metabolic syndrome. After 5 years, workers who initially had one or two risk factors for metabolic disease were 4.6 and 12.7 times, respectively, more likely to develop the condition.

Some studies have found associations between night or shift work and increased food intake, a preference for carbohydrate-rich foods, and alterations in lipid parameters, especially triglyceride levels [23].

Study conducted in China [24] with 26,382 workers (11,783 men and 14,599 women), with total of 9088 shift workers, long-term shift work was associated with metabolic syndrome without adjusting for any confounders factors. In female workers, every 10 years increase in shift work was associated with 10 % (95 % CI: 1 % -20 %) higher odds of metabolic syndrome. Moreover, shift work duration was significantly associated with higher blood pressure levels, higher waist circumference, and increase on glucose levels, all components of metabolic syndrome.

Study conducted by Kawabe in Japanese workers with 3094 subjects in the daytime work group, 73 in the fixed nighttime work group, 1017 in the shift work group and 243 in the day-to-night work group, showed that fixed nighttime and shift work independently contributed to the number of metabolic syndrome components, compared to daytime work [25].

Canuto et al. in a systematic review examined the association between shift work and metabolic syndrome. Eight of out 10 studies found a positive association between shift work and metabolic syndrome, after controlling for socio-demographic and behavioral factors. However, only three studies included sleep duration as a confounder, and these studies presented discordant results. Authors concluded that there was insufficient evidence regarding the association between shift work and prevalent mesbolic syndrome when the confounders are taken into account [26].

### Diabetes mellitus

The strongest evidence linking circadian disruption and type 2 diabetes derives from epidemiological studies which show that shift workers are at increased risk of developing this condition [27, 28].

In a sample of 2860 industry workers followed for 8 years, Morigawa et al. [7] found the incidence of diabetes to be 4.41 per 1000 people/year. The relative risk of diabetes was higher in subjects who worked consecutive night shifts than in those with administrative positions (relative risk: 2.01 after adjusting for all potential confounding factors). The study suggested that shift work is a risk factor for the development of diabetes.

A study performed by Pan et al. [27] compared the association between rotating vs. fixed shifts and type 2 diabetes in 177,000 nurses aged 25–67 years. The risk of type 2 diabetes in participants exposed to rotating shift work for 1–2 years was 5 %. This value increased to 20 % after 3–9 years of rotating shift work, 40 % after 10–19 years, and almost 60 % after 20 years of rotating shifts.

Shift workers of a Japanese manufacturing company were evaluated for the association between shift work and diabetes mellitus according to intensity of work (seasonal or continuous), and adjusted for age, smoking status, frequency of alcohol consumption and status of cohabitation. The odds for diabetes was 0.98 (95 % confidence interval [CI]: 0.28 to 4.81) and 2.10 (95 % CI: 0.77-5,71) among seasonal shift workers and continuous shift workers, respectively, compared to non-shift workers. The risk of diabetes mellitus was more pronounced in continuous shifts workers over 45 years [29]. This discrepancy may be due to circadian rhythm disturbed by a long-term continuous work, possibly causing insulin resistance and weight gain, and hence the type 2 diabetes mellitus [30].

A small study with type 2 diabetes individuals showed that glycemic control did not differ between night vs. day workers, although the night workers had a greater accumulation of visceral fat [31].

In type 1 diabetes patients, diabetes control was affect by shift work. In 296 workers, including 67 (23 %) shift workers, glycemic levels was higher in shift workers than in day worker subjects (HbA1c 9.02 vs. 8.35; P <0.01) [32].

Several experimental studies in both animals and humans have sought to demonstrate the association between sleep disruption or deprivation and the risk of type 2 diabetes [33, 34].

Healthy individuals show reduced glucose tolerance and insulin sensitivity following six consecutive nights of 4 h sleep restriction [15]. In a controlled trial, which sought to determine the interference of circadian disruption on metabolism, 26 healthy adults were divided into two groups and exposed to different sleep restriction protocols. Although total sleep time per day was almost identical between groups, insulin sensitivity decreased significantly after sleep restriction, without a compensatory increase in insulin secretion. In male subjects exposed to circadian misalignment, the reduction in insulin sensitivity and increase in inflammatory activity was twice that of subjects with regular nocturnal sleep [35].

Evidence from human studies suggests that insufficient or poor quality sleep are risk factors for the development and exacerbation of insulin resistance and can increase both appetite and adiposity [36, 37]. A meta-analysis with 10 studies and 107,756 participants assessed the relationship between habitual sleep disturbances and the incidence of type 2 diabetes, and both quantity and quality of sleep predicted the risk of development of diabetes. For short duration of sleep (< or =5–6 h/night), the risk was 1.28, and for long duration of sleep (>8–9 h/night) the risk was 1.48 for incidence of type 2 diabetes and for difficulty in initiating sleep, the risk was 1.57 and for difficulty in maintaining sleep, the risk was 1.84 respectively [38].

During activity and feeding, the blood sugar content is primarily determined by nutrient intake. During rest and fasting, endogenous hepatic glucose production takes place, and glucose levels are maintained within a relatively narrow range. As such, blood glucose homeostasis is also associated with central circadian rhythmicity as well as peripheral oscillators located in regions such as the liver, pancreas, muscles and white adipose tissue [36].

Adipose tissue plays an important role in the endocrine system. In addition to functioning as a fat depot, these tissues play a role in adipokine secretion, which is involved in several physiological pathways, including sugar and energy metabolism. Leptin, adiponectin and visfatin are secreted in a circadian manner [39]. In addition to regulating satiety, leptin also increases energy expenditure and insulin sensitivity. Although it is hypothesized that alterations in the circadian rhythmicity of adipokines may induce insulin resistance, there is as yet no evidence supporting this assumption [36].

### Sleep deprivation

Patel and Hu [40] also revealed an independent association between short sleep duration and weight gain. Gangwisch et al. analyzed data from the First National Health and Nutrition Examination Survey (NHANES I) and found that subjects who slept for less than 6 h a day had higher BMI than those who slept for longer periods [15].

According to the National Health Interview Survey, which followed American adults aged 18 years or older from 1977 to 2009, subjects who slept for less than 5 h a day were 30 % more likely to be overweight and twice as likely to be obese as those who slept for 7 to 8 h a day. Similarly, subjects who slept for 5 to 6 h a day were 20 % more likely to be overweight and 57 % more likely to be obese than those who slept for longer periods of time. On the other hand, a habitual sleep duration longer than 8 h was associated with a 20 % increase in the risk of obesity, but had no impact on the likelihood of being overweight [41].

Sleep deprivation is known to decrease the concentration of leptin (an anorexigenic hormone) and increase levels of ghrelin (an orexigenic neuropeptide) [42]. According to a recent study, a short sleep duration is associated with reduced leptin levels and an increased prevalence of overweight [43]. These findings suggest that these hormonal pathways may be involved in the association between short sleep duration and obesity.

Other studies have also found that sleep deprivation may have an impact on both cognitive and physical performance, induce metabolic alterations such as the suppression of growth hormone production and of the circadian melatonin rhythm, and be associated with the development of metabolic syndrome, type 2 diabetes mellitus, hypertension and immunosuppression [44].

### Circadian rhythm

The circadian rhythm has three main characteristics: it is endogenous, resistant to abrupt changes and may be slow to adapt to changing conditions. The central nervous system (CNS) is the major synchronizer of the human circadian rhythm, and coordinates circadian pacemakers in the brain and peripheral tissues through signals generated in the suprachiasmatic nucleus (SCN) of the hypothalamus which align the circadian period and phase with external stimuli [39].

The functional organization of the circadian system is comprised of three main components: inputs, which can reset the central pacemaker so that it becomes coincident with the external environment (light/dark cycles, social contacts, physical exercise, food); the central pacemaker and peripheral oscillators, present in most peripheral tissues and organs, and even under the command of SCN may occasionally desynchronize the circadian rhythm; and outputs, which are the physiological and behavioral functions, which may also provide feedback by modifying the function of the SCN, oscillators peripheral and suprachiasmatic nucleus (sleep/wakefulness, locomotor activity, endocrine rhythms, body temperature, cardiovascular rhythm, feeding time) [39, 45].

The SCN is directly connected to the retina via the retinal-hypothalamic tract. Signals sent over this pathway synchronize the circadian system to the 24 h day. The SCN then coordinates the remaining oscillators in the brain and peripheral tissues. The light–dark cycle is the most important synchronizer in the central circadian pacemaker [46], while feeding schedules are the most important Zeitgebers, or external synchronizers. As such, abrupt changes in feeding times and others several process, including peripheral temperature control and the sleep-wake cycle, can lead to the desynchronization of circadian rhythms [46, 47].

The causes and consequences of circadian desynchronization may also result from changes in physiological circadian periodicity induced by situational factors such as alterations in light–dark synchronization due to continuous or nocturnal light exposure, frequent snacking, reduced physical activity, nocturnal eating habits or physical activity, daylight savings time and changes in time zone [44]. Since night and shift work tend to affect all aforementioned factors, such schedules are likely to have a significant effect on worker health.

### Desynchronization

The disruption of the internal temporal order is referred to as desynchronization. Repeated desynchronization and resynchronization may alter circadian rhythmicity and cause or accelerate disease, as shown by studies of the association between shift work and several chronic degenerative diseases.

However, despite these investigations, the mechanisms underlying the relationship between circadian desynchronization and obesity have not been fully elucidated. Factors such as frequent snacking, reduced sleep duration and increased exposure to bright light at night time may reduce the perception of internal and external rhythms. Several hypotheses have been proposed to explain the relationship between these factors.

The association among these variables may be mediated by biological processes such as autonomic dysregulation, increased hypothalamic-pituitary-adrenal axis activity or the activation of inflammatory pathways [41].

The autonomic nervous system (ANS) is responsible for controlling the internal circadian clock, especially with regard to abdominal and subcutaneous fat depots [37].

A pineal-hypothalamic-adipocyte hypothesis has also been proposed by Neel and revised by Scott and Grant [48], who suggested that, during longer summer days, pineal melatonin secretion is suppressed and food intake increases, resulting in increased fat deposition in anticipation to winter. These changes result in insulin resistance, increased leptin secretion and adiponectin suppression.

In hibernating animals, the adiponectin suppression and increased leptin secretion are coordinated to induce insulin resistance and decreased appetite in response to decreased food availability. During winter nights, melatonin secretion increases, resulting in increased adipocyte sensitivity to insulin and greater energy availability [48]. In humans, it is as if we are constantly preparing for a long and harsh winter with prolonged food deprivation which, in reality, never takes place.

Several studies have demonstrated that individuals who sleep for shorter periods of time have reduced leptin levels and increased circulating ghrelin, suggesting that sleep deprivation may affect the peripheral regulation of hunger and satiety [42, 49]. Ghrelin is a peptide produced by endocrine cells in the stomach and in the hypothalamus, which plays an important role in the energy balance by stimulating food intake and reducing fat utilization. It is also associated with gastrointestinal functioning and the kinetic properties of the stomach. Unlike most intestinal hormones, ghrelin serum levels increase during fasting and reduce after food intake [50].

An important implication of these findings is that the internal desynchronization of the circadian rhythmicity of satiety-related peptides, especially leptin, may be involved in the imbalance between energy intake and expenditure [42, 43, 49].

Although obesity is a risk factor for the development of type 2 diabetes, recent studies have shown that insufficient sleep may also interfere with glucose metabolism and increase the risk of diabetes regardless of BMI. Sleep restriction may alter the energy balance and induce weight gain by causing both appetite dysregulation and lower calorie expenditure. Excessive weight gain can induce insulin resistance, increasing predisposition to disease and promoting adiposity [27, 34, 51].

### Therapeutic implications

The adaptation to night or shift work depends of several factors, including lifestyle behaviors pertaining to one's family and social life, which usually follow a diurnal pattern. Some studies have found that exposure to bright lights during night-time and the use of melatonin may contribute to circadian synchronization, although this does not apply to all workers [52].

According to a review conducted by Roth [47], the management of shift work disorders must be adjusted to target each specific component of the disease. Circadian misalignment can be treated using melatonin or its agonist ramelteon combined with planned sleep schedules and timed light exposure. Daytime insomnia can be addressed by the use of sedative hypnotics like Zolpidem, Estazolam, Zopiclone and Triazolam, in addition to melatonin. Conversely, Modafinil, Armodafinil, caffeine and light exposure can be used to increase nighttime alertness and avoid or treat somnolence during work hours.

Circadian desynchronization and sleep disorders can also be avoided by having at least 7 h of sleep per 24 h, initiating the main sleep episode immediately after work, taking 30 min to 2 h naps prior to night shifts, napping for 20–30 min during the shift itself to help maintain wakefulness, especially in high-risk occupations, and increasing exposure to bright lights during the first half of the night shift. After the end of the night shift, workers should avoid exposure to bright lights and ensure their bedroom is quiet and dark [53].

Regular shifts and clockwise rotation may also avoid circadian desynchronization and associated health consequences. Haus [44] also recommends that, to avoid or minimize internal desynchronization, workers should be exposed to a maximum of four night shifts, followed by one night off for sleep recovery.

Cortisol levels increase upon waking and decrease over the course of the day. To maintain cortisol rhythmicity, diurnal light exposure should be kept constant, and meal times and exercise should follow a regular schedule [54].

Circadian variations in arterial pressure, which usually fluctuates over the course of the day and decreases at night, must also be monitored in shift workers and considered when prescribing anti-hypertensive medications.

These measures may attenuate the impact of night or shift work on health and must be addressed when organizing shift or night work schedules.

## Conclusions

Shift and night work appear to have a negative effect on worker health, possibly due to its impact on sleep-wake cycles, eating and exercise habits, thermogenesis, hormone secretion, and blood pressure levels [39, 54, 55].

By identifying the association between shift characteristics and their impact on the body, we can find ways to minimize the health burden of shift work, so that societal demands can be met without compromising worker health. The comprehension of the mechanisms responsible for the effects of shift work on the body may contribute to the development of weight reduction strategies or elucidate ways to avoid weight gain altogether based on low-calorie diets, personalized health care and nutritional orientations, in addition to physical exercise programs which can be adapted to the worker's routine [45, 52, 54].

## Abbreviations

SAH: Systemic arterial hypertension
CNS: Central nervous system
SCN: Suprachiasmatic nucleus
LDL: Low-density lipoprotein
BMI: Body mass index
ANS: Autonomic nervous system
